# Comparative population genetic structure of redbelly tilapia (*Coptodon zillii* (Gervais, 1848)) from three different aquatic habitats in Egypt

**DOI:** 10.1002/ece3.3586

**Published:** 2017-11-15

**Authors:** Taha Soliman, Walid Aly, Reda M. Fahim, Michael L. Berumen, Holger Jenke‐Kodama, Giacomo Bernardi

**Affiliations:** ^1^ Okinawa Institute of Science and Technology Graduate University (OIST) 1919‐1 Tancha, Onna‐son Kunigami‐gun Okinawa Japan; ^2^ National Institute of Oceanography and Fisheries Cairo Egypt; ^3^ Division of Biological and Environmental Sciences and Engineering Red Sea Research Center King Abdullah University of Science and Technology Thuwal Saudi Arabia; ^4^ Department of Ecology and Evolutionary Biology University of California Santa Cruz Santa Cruz CA USA

**Keywords:** aquaculture introduction, genetic diversity, mtDNA, redbelly tilapia, saltwater adaptation

## Abstract

Recently, tilapia have become increasingly important in aquaculture and fisheries worldwide. They are one of the major protein sources in many African countries and are helping to combat malnutrition. Therefore, maintenance and conservation genetics of wild populations of tilapia are of great significance. In this study, we report the population genetic structure and genetic diversity of the redbelly tilapia (*Coptodon zillii*) in three different Egyptian aquatic environments: brackish (Lake Idku), marine (Al‐Max Bay), and freshwater (Lake Nasser). The habitat differences, environmental factors, and harvesting pressures are the main characteristics of the sampling sites. Three mitochondrial DNA markers (COI: cytochrome oxidase subunit I; the D‐loop; CYTB: cytochrome b) were used to assess population structure differences among the three populations. The population at Lake Nasser presented the highest genetic diversity (*H*_*d*_ = 0.8116, *H *=* *6), and the marine population of Al‐Max Bay the lowest (*H*_*d*_ = 0.2391, *H* = 4) of the combined sequences. In addition, the phylogenetic haplotype network showed private haplotypes in each environmental habitat. Results presented here will be useful in aquaculture to introduce the appropriate broodstock for future aquaculture strategies of *C. zillii*. In addition, evidence of population structure may contribute to the management of tilapia fisheries in Egyptian waters.

## 1 INTRODUCTION

The native African tilapiine fish belonging to the family Cichlidae (Teleostei, Perciformes) are widely distributed among tropical, subtropical, and temperate zones (Fryer & Iles, 1972; Pillay, 1990). A recent revision of African cichlids recognized 20 haplotilapiine genera and nine tribes based on phylogenetic relationships and morphological characters (Dunz & Schliewen, 2013). Three genera of tilapiine cichlids, *Coptodon* Gervais, 1853, *Oreochromis* Günther, 1889, and *Sarotherodon* Rüppell, 1854, are abundant in Egyptian waters. The Nile tilapia, *Oreochromis niloticus,* together with *O. aureus*,* Coptodon zillii*, and *Sarotherodon galilaeus* are important aquaculture and/or fisheries species in Egypt (El‐Sayed, 2004, 2006). Consequently, tilapias are economically important, comprising the majority (58.54%) of total Egyptian fish production, where 7.29% are harvested from natural resources (River Nile and other Egyptian inland waters) and 51.25% are produced by aquaculture (GAFRD 2014). Additionally, Egypt is the second largest producer of farmed tilapia in the world, and it supplies 62%–66% of all African tilapia (El Naggar & Jiddou, 2013; FAO 2016).

Adaptation of tilapia to different environmental conditions (wide range of temperatures, salinity, and pH) has been reported in many studies (e.g., Anthoni, Børresen, Christophersen, Gram, & Nielsen, 1990; Chervinski & Hering, 1973; Chervinski & Zorn, 1974; Cnaani, Gall, & Hulata, 2000). Most tilapia species are euryhaline, tolerating a wide range of salinities. For example, *Coptodon zillii* can live in salinities as low as 0‰ and as high as 45‰ (Chervinski & Hering, 1973; Chervinski & Zorn, 1974). The metabolites taurine (2‐aminoethanesulfonic acid) and trimethylamine oxide (TMAO) are important in osmoregulation and other physiological functions of marine fishes (Trachtman, del Pizzo, & Sturman, 1990; Vanwaarde, 1988). Anthoni et al. (1990) reported high levels of TMAO and taurine in tilapia, which may facilitate their adaptation to different environments, as observed in other marine species.

Genetic tools have proven useful in fisheries and aquaculture management (Hedgecock, 1987; Ovenden, Berry, Welch, Buckworth, & Dichmont, 2015), as well as taxonomy and species identification (Nagl et al., 2001), estimation of population distributions (Hedgecock, 1987), and larval dispersal and migration (Lowe & Allendorf, 2010). Moreover, several studies focused on population genetic structure and genetic diversity of tilapias under different environmental conditions using mtDNA and microsatellites (Hassanien & Gilbey, 2005; Szitenberg, Goren, & Huchon, 2012).

*Coptodon zillii*, known previously as *Tilapia zillii*, is one of the widely distributed tilapia species found throughout Egyptian waters. It has been recorded in freshwater and brackish lakes (Alsayes, Shehata, & Soliman, 2008), lagoons (Chervinski, 1972), the Nile River (El‐Bokhty & El‐Far, 2014), the Mediterranean Sea (Akel & Moharram, 2007; Chervinski, 1987), and the Red Sea (Bayoumi, 1969). Lake Idku, Al‐Max Bay, and Lake Nasser are important sites for Egyptian fisheries.

The goal of this study was to assess population structure in *C. zillii* by comparing its genetic characteristics in three different environments. Three Egyptian aquatic habitats differ in fish harvesting pressure and habitat characteristics. For example, tilapia is not a target fish for fishermen in Al‐Max Bay, but it is the main target in Lake Nasser and Lake Idku. In addition, the impact of anthropogenic activities (enclosed by large fishermen communities and fish farms) and sea levels affected the demographic population structure in Lake Idku and the north Delta lakes (Malm & Esmailian, 2012). However, Lake Nasser is surrounded by small fishing communities, and fishing pressures are influenced by factors such as high temperatures and fish transportation. Additionally, the Aswan High Dam isolates Lake Nasser from other Egyptian aquatic environments. Therefore, we expected some differences in population structure and genetic diversity among *C. zillii*. The results of this study are the first genetic study on the Egyptian populations of *C. zillii* based on three mtDNA markers (COI, D‐loop, and CYTB), and these data will be useful to assess population structure, which is needed for aquaculture and fisheries management.

## 2 MATERIALS AND METHODS

### 2.1 Sampling

**Figure 1:**
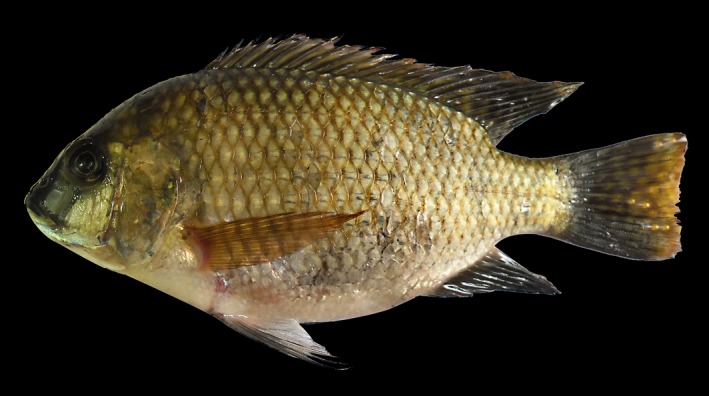
Image of fresh specimen (15 cm) of *Coptodon zillii* (Gervais, 1848)

**Figure 2:**
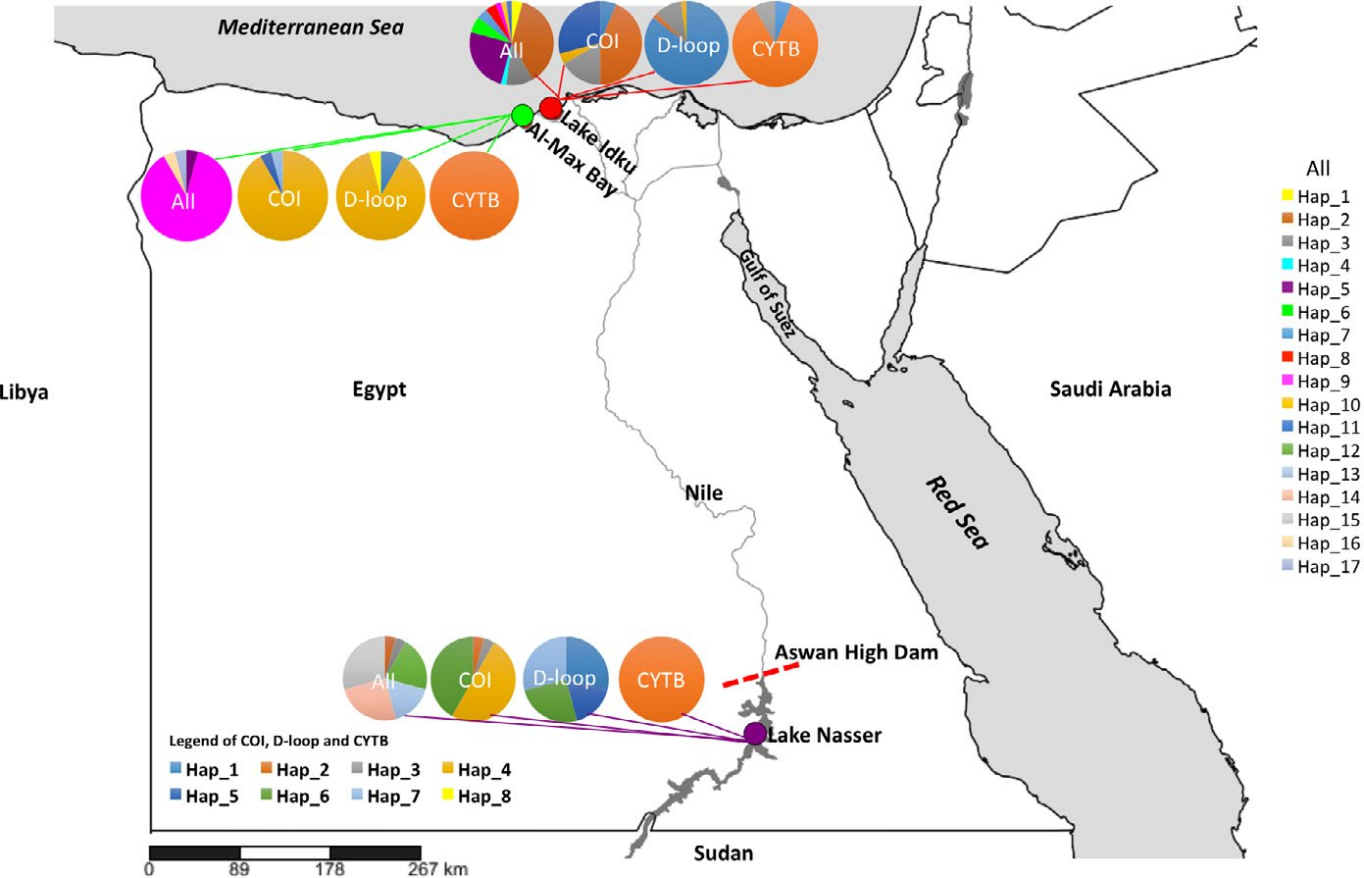
Map showing sampling sites of *Coptodon zillii* from three different types of Egyptian aquatic habitats. The pie charts represent the distribution of haplotypes of combined sequences of all loci (All), *cytochrome oxidase subunit I* (COI), *D‐loop* region, and *cytochrome b* (CYTB), as defined by Figure 2 and Table 1

Ninety‐six individuals of *C. zillii* (Figure 1) were collected by trammel net from three different locations [Lake Idku (brackish), Al‐Max Bay (marine), and Lake Nasser (fresh)] in October and November 2015 (Table 1 and Figure 2). Lake Idku (31.2482 N, 30.2013 E) is one of the brackish northern lagoons that connect to the Mediterranean Sea (Ali & Khairy, 2016), and Al‐Max Bay (31.1383 N, 29.8144 E) is located on the coast of the Mediterranean Sea, west of Alexandria (El‐Saharty, 2013) (Figure 2). Lake Nasser (23.2098 N, 32.8118 E), located in the southern part of Egypt, is one of the largest human‐made reservoirs (Rashed, 2001). Individuals of *C. zillii* were identified using morphological characteristics (Dunz & Schliewen, 2010, 2013); muscle tissue (~ 4 g) from each specimen was preserved in 99.5% ethanol.

**Table 1:** Genetic diversity indices and neutrality test (Fu's *F*_*S*_) of *Coptodon zillii* from different Egyptian aquatic habitats

Location	Salinity (%)	*N*	*H*	*H*_*d*_	5	π	Fu's *F*_*s*_	Fu's *F*_*s*_ (*p*‐values)
COI								
Lake Idku	1.5–3	48	5	0.7048	5	0.0025	1.3539	0.7810
Al‐Max Bay	32	24	3	0.1630	4	0.0006	−0.4623	0.2530
Lake Nasser	<0.5	24	4	0.5978	4	0.0019	0.7482	0.6760
D‐loop								
Lake Idku	1.5–3	48	4	0.2642	4	0.0011	−1.4012	0.1610
Al‐Max Bay	32	24	3	0.2355	4	0.0014	0.1407	0.4220
Lake Nasser	<0.5	24	4	0.7717	5	0.0046	1.9357	0.8480
CYTB								
Lake Idku	1.5–3	48	3	0.2651	2	0.0006	−0.8041	0.2130
Al‐Max Bay	32	24	1	0.0000	0	0.0000	0.0000	N.A.
Lake Nasser	<0.5	24	1	0.0000	0	0.0000	0.0000	N.A.
Combined								
Lake Idku	1.5–3	48	11	0.7917	11	0.0015	−2.1651	0.1780
Al‐Max Bay	32	24	4	0.2391	8	0.0006	0.1130	0.4970
Lake Nasser	<0.5	24	6	0.8116	9	0.0021	1.4736	0.8170

(*N*) Sample size, (*H*) number of haplotypes, (*H*_*d*_) haplotype diversity, (*S*) number of segregating sites, (π) nucleotide diversity; *p < 0.05*.

### 2.2 DNA extraction and sequencing

Total genomic DNA was extracted from muscle tissue (~25 mg) using a DNeasy Blood & Tissue extraction kit (Qiagen, Tokyo, Japan) following the manufacturer's protocol. PCRs were conducted using three primers for three regions of mtDNA: the D‐loop (noncoding) control region using universal primers CR‐A, 5′‐TTCCACCTCTAACTCCCAAAGCTAG ‐3′ and CR‐E, 5′‐CCTGAAGTAGGAACCAGATG ‐3′ (Lee, Conroy, Howell, & Kocher, 1995); Cytochrome oxidase subunit I (COI) FishF1, 5′‐TCAACCAACCACAAAGACATTGGCAC‐3′ and FishR1, 5′‐TAGACTTCTGGGTGGCCAAAGAATCA‐3′ (Ward, Zemlak, Innes, Last, & Hebert, 2005); and Cytochrome b gene (CYTB) L14724 5′‐CGAAGCTTGATATGAAAAACCATCGTTG‐3′ and H15149 5′‐AAACTGCAGCCCCTCAGAATGATATTTGTCCTCA‐3′ (Irwin, Kocher, & Wilson, 1991).

PCR amplifications were conducted in a 20 μl total volume containing 10–20 ng/μl of template genomic DNA, 0.5 μM of forward and reverse primers, and 10 μl of HotStarTaq Master Mix Kit (Qiagen, Tokyo, Japan) in deionized water. PCR cycling was performed in an Eppendorf thermocycler (Eppendorf AG, Hamburg, Germany) using an initial denaturation step at 95°C for 15 min, followed by 35 cycles of denaturation at 94°C for 45 s, optimum annealing (*Tm*_D‐loop_ = 52°C and *Tm*_COI and CYTB_ = 50°C) for 45 s, and extension at 72°C for 1 min and a last extension step at 72°C for 10 min. The amplification products were examined by electrophoresis (1.5% agarose gel). They were purified with Exonuclease I and Alkaline Phosphatase Shrimp (Takara, Japan) and were incubated at 37°C for 20 min, followed by deactivation at 83°C for 30 min. Cleaned PCR products were sequenced using an ABI 3730 Genetic Analyzer at Fasmac Co., Kanagawa, Japan. Consensus sequences were edited and aligned using Geneious^®^ 8.1.9 (Kearse et al., 2012).

### 2.3 Data analysis

We used the software DnaSP (Librado & Rozas, 2009) to compute the number of haplotypes (*H*), haplotype diversity (*H*_*d*_), the number of segregating sites (*S*), and nucleotide diversity (π). Neutrality tests based on Fu's *F*_S_ were performed in the program Arlequin, version 3.5.2.2 (Excoffier & Lischer, 2010). The best‐fit model of DNA sequence evolution was tested and estimated using MEGA version 7.0 (Kumar, Stecher, & Tamura, 2016). The Akaike information criterion (AIC) indicated that Jukes–Cantor (JC) was the best‐fit model for all markers (D‐loop, COI, CYTB, and combined sequences) of this study. Pairwise Φ_ST_ was calculated using Arlequin version 3.5.2.2 (Excoffier & Lischer, 2010) with 10,000 permutations, and simultaneous test corrections were adjusted using the modified false discovery rate (FDR) method (Narum, 2006). PopART program version 1.7 (http://popart.otago.ac.nz/) was used to build the phylogenetic relationship among haplotypes of all markers (D‐loop, COI, CYTB, and combined sequences) between geographic sampling sites using median‐joining (MJ) network algorithm (Bandelt, Forster, & Rohl, 1999). The expected and observed mismatch distribution of *C. zillii* was estimated using DnaSP version 5.10.1 (Librado & Rozas, 2009) based on COI and D‐loop sequence data. Mantel's test was performed for COI and D‐loop sequence data in order to estimate the isolation by distance (IBD) among populations. The correlation between *Φ*_ST_ and geographic distance (log) was estimated with 20,000 randomizations using IBDWS version 3.23 (Jensen, Bohonak, & Kelley, 2005).

## 3 RESULTS

Sequence fragment lengths in the 96 analyzed *Coptodon zillii* were 657 bp of cytochrome oxidase subunit I (COI), 412 bp of D‐loop, and 439 bp of Cytochrome b (CYTB). In total, 8, 7, 3, and 17 mtDNA haplotypes were obtained from genes D‐loop, COI, CYTB, and combined sequences for the three populations of *C. zillii*, respectively (Table 1). Haplotype sequence data from this study were deposited in GenBank under accession numbers KY465475 ‐ KY465492. Distributions and frequencies of haplotypes for the studied locations (Lake Idku, Al‐Max Bay, and Lake Nasser) in each genetic marker are shown in Table 1 and Figure 2. Haplotype diversities among all genetic markers and combined sequences were low, ranging from 0.0000 to 0.2391 in Al‐Max Bay. The freshwater population of Lake Nasser showed higher haplotype diversity and nucleotide diversity based on the D‐loop region (*H*_*d*_ = 0.7717; π = 0.0046) and combined sequences (*H*_*d*_ = 0.8116; π = 0.0021). However, haplotype diversity and nucleotide diversity of the brackish water population in Lake Idku were higher than in the freshwater population based on COI (*H*_*d*_ = 0.7048; π = 0.0025) and lower using other markers (Table 1). Among all populations, CYTB indicated significantly lower haplotype diversity (0.0000–0.2651) compared to other genes studied here (D‐loop and COI). Interestingly, results based on CYTB showed low or monomorphic results of the genetic indices for *C. zillii* among all populations. This gene has some limitations as a phylogenetic marker, and it fails to resolve the lower genetic relationships in cichlid fishes because of its slow mutation rate (Farias, Orti, Sampaio, Schneider, & Meyer, 2001). Therefore, CYTB seems to be a poor marker to study the population genetics of *C. zillii*. Therefore, we focus primarily on results based on the D‐loop and COI markers, which show variability consistent with previous studies (e.g., Wu & Yang, 2012).

The results of the neutrality test of Fu's *F*_*S*_, based on the infinite site model and its *p*‐values, are shown in Table 1. Among all populations, Fu's *F*_*S*_ *p*‐values <0.02 showed insignificant difference among all markers of this study. Additionally, Fu's *F*_*S*_ showed negative values in Al‐Max Bay (COI = −0.4623) and Lake Idku (D‐loop = −1.4012; CYTB = −0.8041; combined = −2.1651). Overall, neutrality tests showed no signs of expansion for any marker in any site.

**Figure 3:**
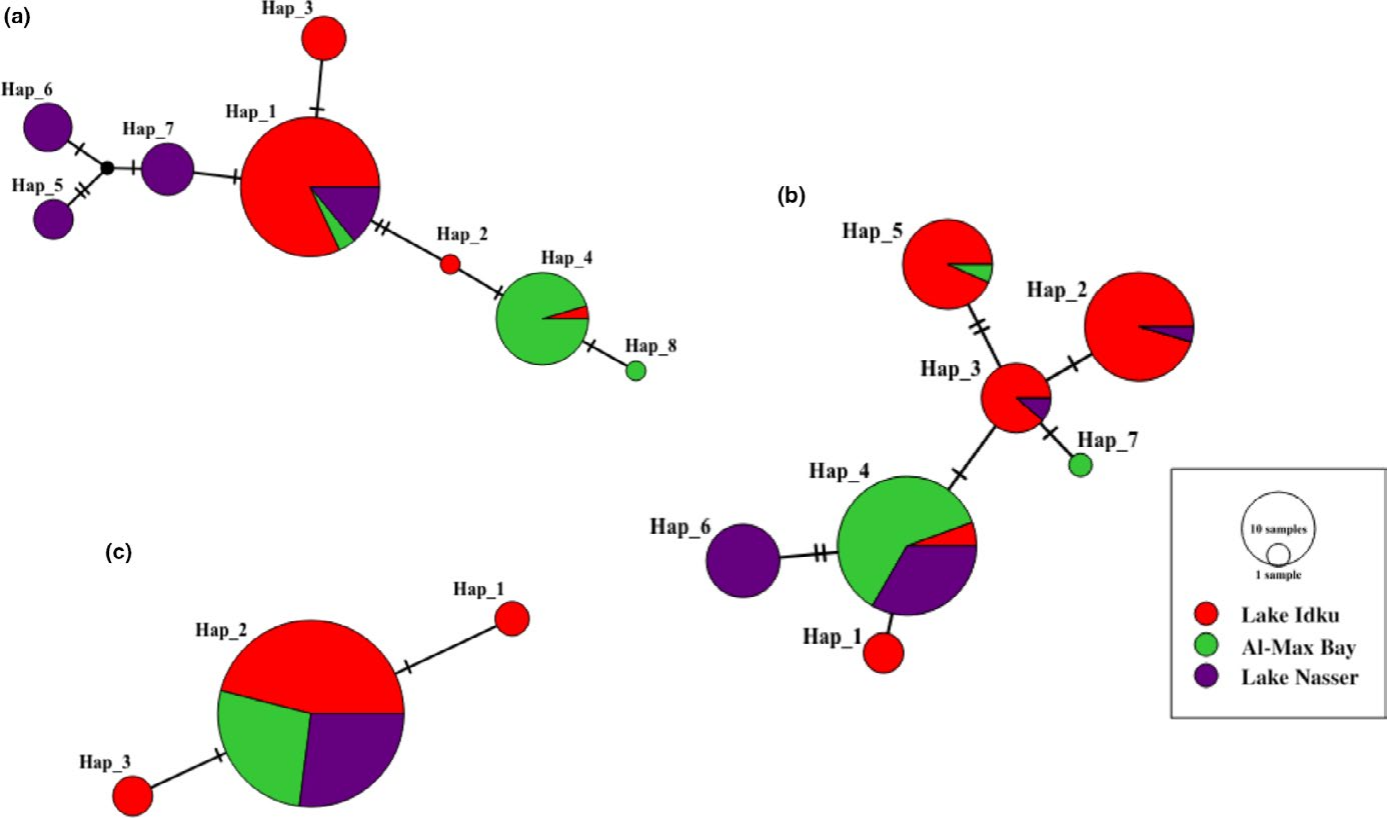
Median‐joining haplotype network inferred from mtDNA (a) *D‐loop* region, (b) *cytochrome oxidase subunit I* (COI) and (c) *cytochrome b* (CYTB) sequences of *Coptodon zillii* from different Egyptian localities. Each circle represents a different haplotype. The size of a circle is proportional to the frequency of each haplotype

**Figure 4:**
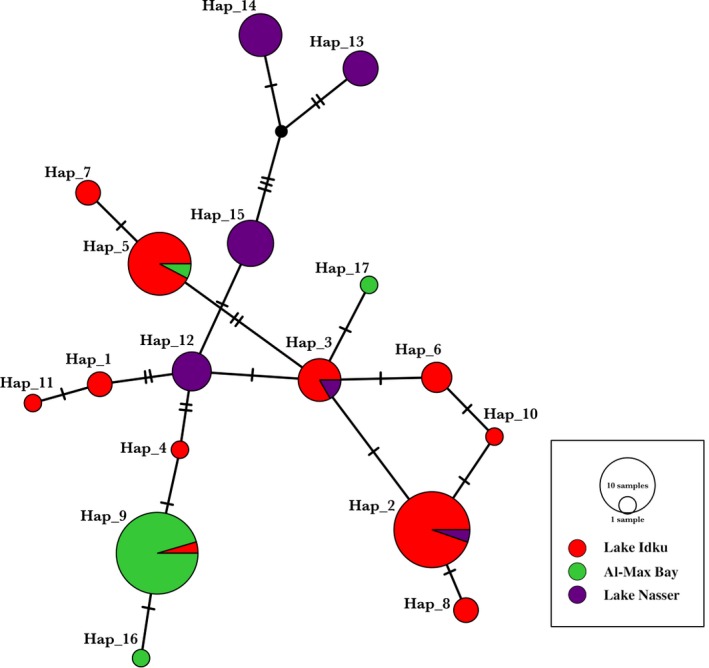
Median‐joining haplotype network inferred from mtDNA combined sequences for all loci (*D‐loop* region, *cytochrome oxidase subunit I,* and *cytochrome b)* of *Coptodon zillii* from different Egyptian habitats. Each circle represents a different haplotype. The size of a circle is proportional to the frequency of each haplotype

Phylogenetic networks of the haplotypes were identified using the median‐joining algorithm for 8,7,3, and 17 haplotypes of D‐loop, COI, CYTB, and joined sequences, respectively (Figures 3 and 4). The haplotype network had relatively star‐like shape in two of the three markers with some unique haplotypes at the edges of each network (Figure 3a and b). The Hap_1 haplotype was dominant in all three sites. According to the D‐loop haplotype network, the Hap_4 haplotype was dominant in Lake Idku and Al‐Max Bay (Figure 3a). In addition, the haplotype network of the COI genetic marker presented fewer unique haplotypes (Hap_1, Hap_6, and Hap_7 among all sites (Figure 3b). However, the lowest number of haplotypes was obtained using the CYTB genetic marker (Figure 3c). The joined sequences (i.e., using all markers combined) showed more distinction among the three sites, with 7, 2, and 4 unique haplotypes from Lake Idku, Al‐Max Bay, and Lake Nasser, respectively (Figure 4). Overall, the haplotypes networks according to two variable markers (D‐loop and COI), marine, and brackish sites show very different haplotype distributions, and that in the freshwater site, almost all haplotypes are present. However, the freshwater site presents several private combined haplotypes, and none in common with the marine site (because they present very different D‐loops).

Pairwise Φ_ST_ values were estimated to assess the population genetic structure of *C. zillii* among the three aquatic environments (Table 2). Two markers (D‐loop and COI) of mtDNA presented significant (*p* < 0.05) Φ_ST_ values. Pairwise *Φ*_ST_ values ranged from 0.3796 to 0.8657 for the D‐loop and from 0.2943 to 0.5439 for COI (Table 2). The CYTB genetic marker showed nonsignificant (*p* < 0.05) Φ_ST_ values among all populations (Table 2). In addition, the concatenated sequences showed significant Φ_ST_ values among all populations as shown by D‐loop and COI markers (Table 2).

**Table 2:** Φ_ST_ pairwise inferred from mtDNA COI (below diagonal), D‐loop (above diagonal), CYTB (below diagonal), and combined sequences (above diagonal) among different Egyptian populations of *Coptodon zillii*

	Lake Idku	Al‐Max Bay	Lake Nasser
COI and D‐loop			
Lake Idku	0.0000	**0.8657**	**0.5017**
Al‐Max Bay	**0.4062**	0.0000	**0.3796**
Lake Nasser	**0.5439**	**0.2943**	0.0000
CYTB and combined			
Lake Idku	0.0000	**0.6933**	**0.2332**
Al‐Max Bay	0.0853	0.0000	**0.4329**
Lake Nasser	0.0853	0.0000	0.0000

Significant values in bold (*p < 0.05*).

The mismatch distribution analysis for both mtDNA markers (COI and D‐loop) revealed multimodal pattern among the observed distribution values (Appendix S1). The isolation by distance (IBD) was calculated for the three populations and indicated insignificant correlations (COI: *r* = −0.3750, *p* = 0.6430; D‐loop: *r* = −0.7285, *p* = 0.8140) between Φ_ST_ and geographic distance among all populations (Appendix S2).

## 4 DISCUSSION

We found clear genetic population structure in the redbelly tilapia, *Coptodon zilli*, among three different types of Egyptian aquatic habitats (freshwater, brackish, and marine) using three mtDNA markers (COI, D‐loop, and CYTB). The genetic isolation of the freshwater population (Lake Nasser) from the brackish and marine populations might be due to anthropogenic factors such as the Aswan High Dam (Figure 2) and fishing activities, as well as habitat differences (Hassanien & Gilbey, 2005). In the Dead Sea system (the Kishon River and Jordan River), hypersaline waters have been shown to create biological barriers for *C. zilli* populations (Szitenberg et al., 2012). A similar pattern of habitat‐driven genetic isolation was reported in Nile tilapia (*Oreochromis niloticus*) (Hassanien & Gilbey, 2005). As aquaculture and other anthropogenic activities continue to expand, it will be important for management entities to consider the impacts of these activities on genetic diversity and connectivity of target species.

The saltwater population (Al‐Max Bay) presented the lowest genetic diversity (0.1630_COI_; 0.2355_D‐loop_; 0.2391_Combined_) based on both genetic markers (COI and D‐loop). Founder effects, predation, fishing pressure, and pollution (e.g., low oxygen levels and eutrophication) may all contribute to the observed low genetic diversity (Lucas, Baras, Thom, Duncan, & Slavík, 2001). Other hypotheses may help explain these results: 1) Al‐Max Bay population arose from inbred individuals escaped from nearby aquaculture facilities or that transferred through water discharge from drains in the surrounding areas. 2) The Al‐Max Bay population is comprised of only a limited subset of genotypes, specifically genotypes that can tolerate the bay's relatively high salinity, similar to Dead Sea populations compared to nearby freshwater populations (Szitenberg et al., 2012). Fu's *F*_*S*_ test of neutrality showed negative values in Lake Idku (D‐loop = −1.4012; combined = −2.1651) indicating an excess number of alleles and contemporary population expansion. This pattern of population expansion may be due to demographic changes in Lake Idku (a semiclosed system). The demographics of Lake Idku are changing rapidly because the lake lost about 319.3 km^2^ of surface area between years 1800 and 2010 (Shawer & Ibrahim, 2010). The reduced area (~27%) of the lake is mainly caused by human impacts in the surrounding area (Malm & Esmailian, 2012; Shawer & Ibrahim, 2010). However, Lake Nasser (the second largest artificial lake in Africa) presented positive values of Fu's *F*_*S*_ test, indicating overdominant selection or a recent bottleneck in its *C. zillii* population. It might be that the population of *C. zillii* in Lake Nasser has a stable overdominant equilibrium. Additionally, two characteristics of Lake Nasser affect population structure of tilapia in all Egyptian waters: 1) the Aswan High Dam was built in 1970, creating a barrier to exchange between populations on either side of the dam, and 2) the presence of various dendritic inlets (Khors) or side projections of the main lake (Ryder & Henderson, 1975; Van Zwieten, Béné, Kolding, Brummett, & Valbo‐Jorgensen, 2011).

This study has clear implications for the fisheries management and aquaculture sectors for *C. zillii* of Egyptian waters. Recently, in some countries, such as Ethiopia, construction of new dams on the Nile River has begun, and these are likely to further affect the diversity, distribution, and population structure of aquatic species in Lake Nasser and other Nile River populations. Therefore, the present study may serve as an important scientific resource for future studies to monitor the influence of existing and new dams. Such structures appear to have the ability to influence connectivity and subsequent genetic structure of the target populations. Our preliminary findings may support fisheries managers, for example, to understand the population structure and diversity of *C. zillii* in Nile waters. Loss of genetic diversity may lead to reduced resilience in the face of unforeseen potential ecological pressures, such as the emergence of diseases or susceptibility to pollutants. We hope that regional authorities will consider and/or monitor population diversity as part of adaptive management plans in this commercially important species.

## CONFLICT OF INTEREST

None declared.

## AUTHOR CONTRIBUTION

Taha Soliman designed and performed the experimental work such as sample collection, DNA extraction, and PCRs, performed data analysis, wrote, and reviewed the manuscript. Walid Aly and Reda M. Fahim collected the samples, designed experimental work, and reviewed the manuscript. Michael L. Berumen analyzed data and reviewed the manuscript. Holger Jenke‐Kodama provided the kits and chemicals and reviewed the manuscript. Giacomo Bernardi designed the experimental work, analyzed data, and reviewed the manuscript.
