# Supplementary figures and images for: Comparative population genetic structure of redbelly tilapia (Coptodon zillii (Gervais, 1848)) from three different aquatic habitats in Egypt
Source: Ecol Evol. 2017 Nov 15;7(24):11092–9. doi: 10.1002/ece3.3586 (PMC5743693; doi:10.1002/ece3.3586)

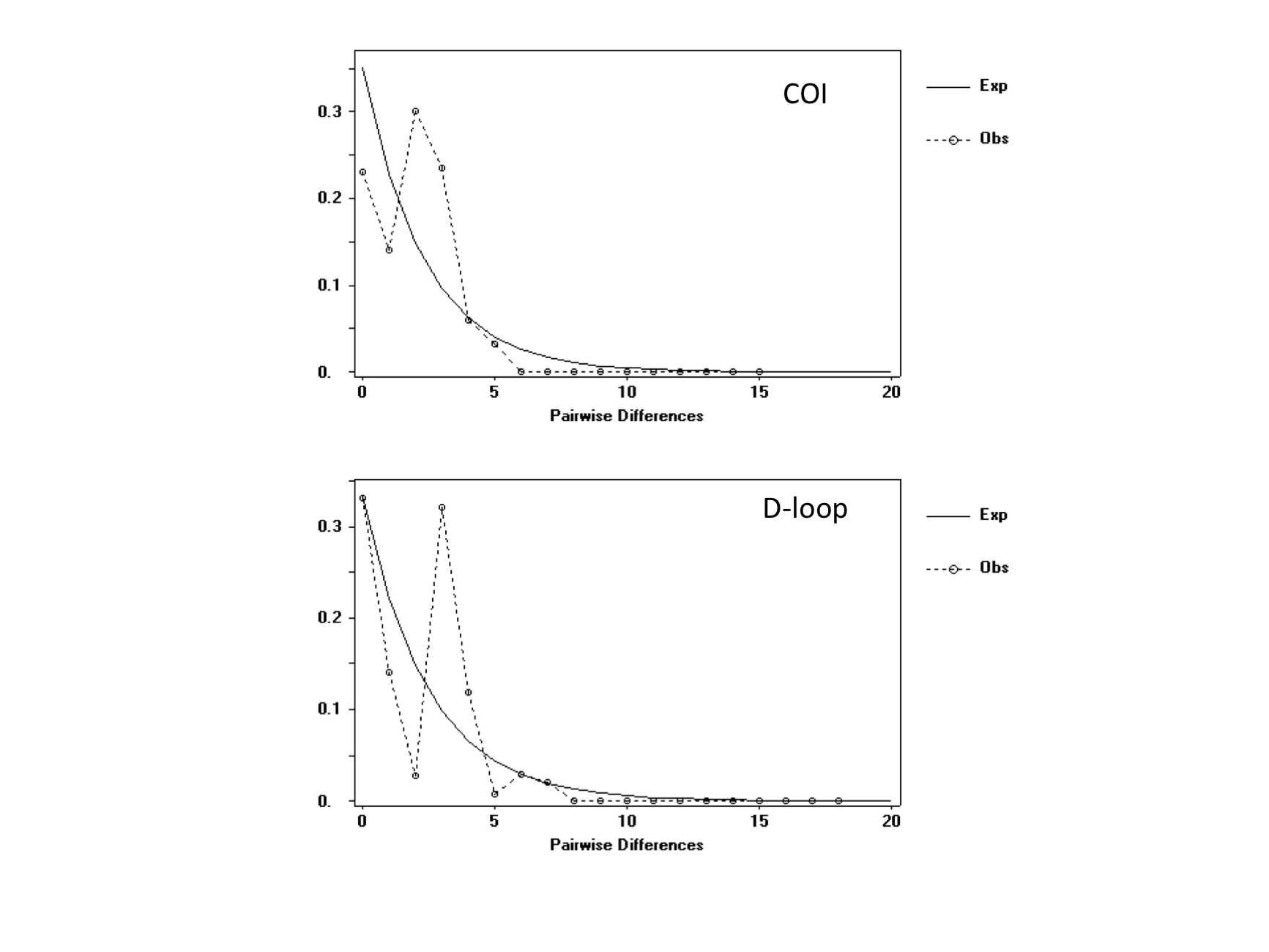

Supplement: Supplementary file 1 [file ECE3-7-11092-s001.tiff]

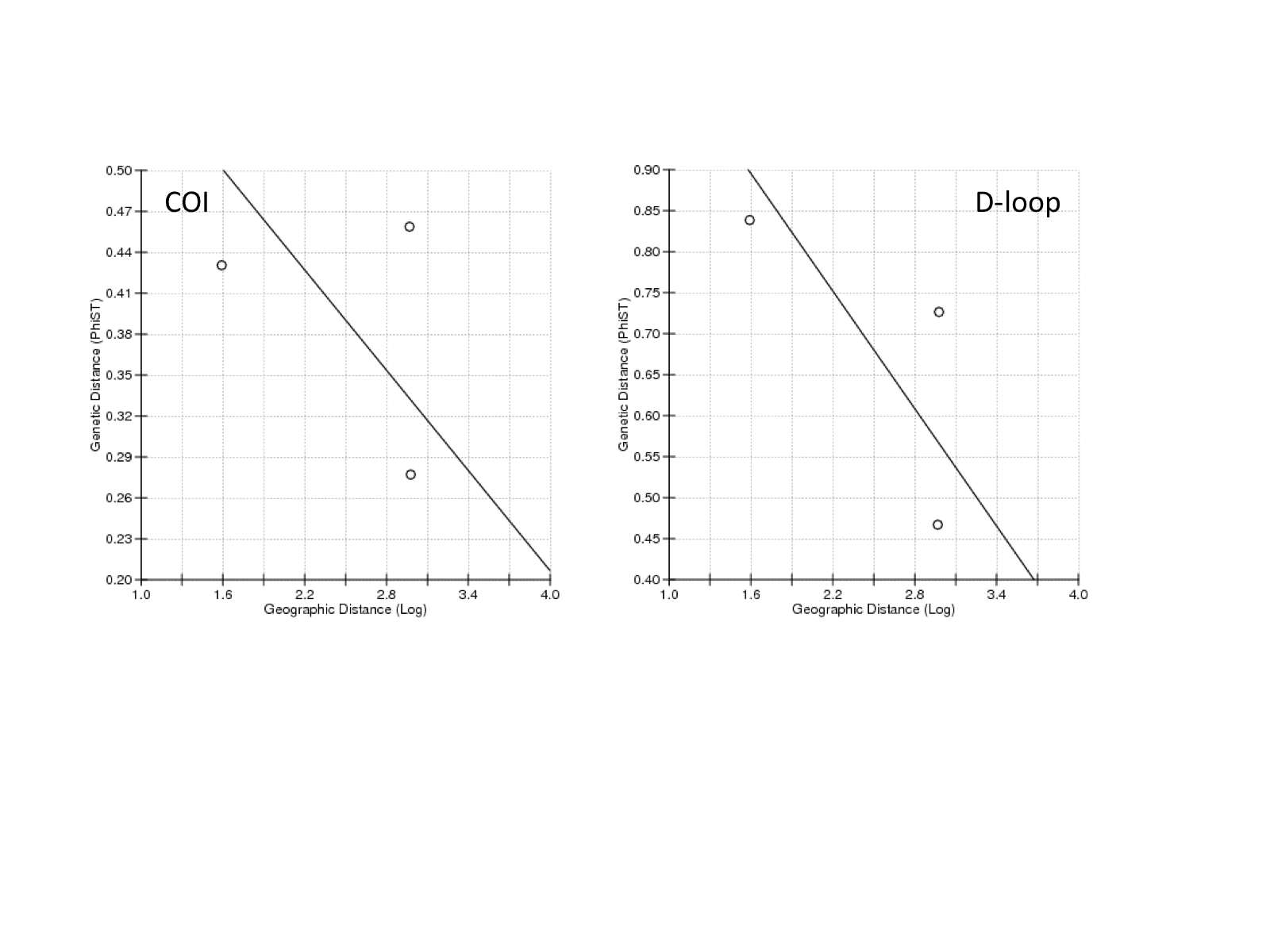

Supplement: Supplementary file 2 [file ECE3-7-11092-s002.tiff]
